# Canine Leishmaniosis Control through the Promotion of Preventive Measures Appropriately Adopted by Citizens

**DOI:** 10.1155/2020/8837367

**Published:** 2020-06-27

**Authors:** Giulia Simonato, Erica Marchiori, Federica Marcer, Silvia Ravagnan, Patrizia Danesi, Fabrizio Montarsi, Carlo Bononi, Gioia Capelli, Mario Pietrobelli, Rudi Cassini

**Affiliations:** ^1^Department of Animal Medicine, Production and Health, University of Padova, Viale dell'Università 16, 35020 Legnaro (PD), Italy; ^2^Istituto Zooprofilattico Sperimentale delle Venezie, Viale dell'Università 10, 35020 Legnaro (PD), Italy

## Abstract

Canine leishmaniosis (CanL) is a disease caused by the protist *Leishmania infantum* and transmitted to dogs by sand fly (Diptera: Phlebotominae) bites. In 2005, a new autochthonous focus of CanL was recognised in the southern part of Euganei hills (northeastern Italy). In subsequent years, this outbreak was monitored, testing dogs and evaluating sand fly population. Moreover, dog owners were sensitized on the adoption of preventive measures, thanks to the collaboration of local administration, health authorities, and private veterinarians. This study includes serological tests on dogs, questionnaires submitted to dog owners regarding the use of preventive measures on their animals, and the evaluation of sand fly abundance. Data collected were statistically compared with those of previous years. The canine seroprevalence was significantly lower than that recorded at the beginning of the outbreak, despite the fact that sand fly abundance did not significantly decrease. In addition, most of the dog owners declared using regularly the topical insecticides on their dogs during the sand fly season. This experience demonstrated that a collaborative approach among scientific researchers, local authorities, and private veterinarians can achieve excellent results in the management of a leishmaniosis outbreak.

## 1. Introduction

Leishmaniases are diseases caused by different species of the genus *Leishmania*, with a huge impact on human population at global level. Among *Leishmania* species causing disease in humans, most have a zoonotic nature and different species of animals are playing an important role in the parasite life cycle [1]. In the Mediterranean basin, the disease is caused by *Leishmania infantum* and transmitted through infected phlebotomine sand fly bite, mainly of the genus *Phlebotomus* [2, 3]. The disease in dogs, which are considered the main reservoir of the parasite for humans, is known as canine leishmaniosis (CanL) [1].

Most of the territory of central and southern Italy is considered endemic [4], whereas the parasite was absent from northern Italy up to the mid ‘90s, when new autochthonous outbreaks have been documented in the last 20 years [5–8]. Due to the fatal aspects in dogs and to its zoonotic role, CanL has to be monitored and managed both in endemic and new areas of spreading [9].

In addition to the treatment of sick dogs, the main action to be implemented is the prevention of sand fly bite of all the exposed dogs, whether they are sick, infected, or in good health, to limit the pathogen circulation. A good level of prevention can be achieved when dogs are kept indoors during the sand fly season from sunset to sunrise [10, 11], but the most effective and common action consists in the use of topical insecticides on dogs with proven activity against sand flies [9, 11]. Many formulations, which have been evaluated under laboratory and field conditions, i.e., spot-on, collars, and spray, are now commercially available [12–16]. In endemic areas, the vaccination is another control measure to prevent clinical leishmaniosis. In Europe, two types of vaccine are now available, but they confer a partial protection against the disease [17, 18]. Therefore, their adoption is suggested in addition to topical insecticides.

This study describes the final outcome of an outbreak of CanL in the southern part of Euganei hills (northeastern Italy), where the first autochthonous case was recognised in 2005 in a small village (Calaone, Baone municipality, Padova province), and subsequent sensitization activities were conducted to promote the use of preventive measures [19], involving the local administration, private veterinarians, and local health authorities. Citizens were informed on the disease during the one-day sampling campaigns conducted in May 2006, June 2007, and May 2010, and thanks are due to specific meetings organized for the dog owners and overall population by the local administration in October 2006, May 2008, and June 2011, with interventions from experts in Public Health, Entomology, and Parasitology. Dog owners were invited to adopt preventive measures, including specifically the use of effective topical insecticides commercially available (i.e., deltamethrin-impregnated collar, permethrin, and permethrin/imidacloprid spot-ons) during the sand fly activity period (June-September in the study area). Moreover, in 2009, the Baone municipality provided local dog owners with about 100 deltamethrin-impregnated collars free of charge to be adopted in the following summer season. The aim of this study was to evaluate the level of appropriate adoption of preventive measures by dog owners of this area and to assess the efficacy of the intervention comparing the current epidemiological situation with that described in the past.

## 2. Materials and Methods

### 2.1. Study Area

The study was conducted in Calaone (Baone municipality, Padova province), a small village located in the southern part of Euganei hills (45°14′58^″^N-11°39′54 ^″^E), characterized by Mediterranean climate. Calaone is located mostly 100 m above sea level (a.s.l.), with an average altitude of 223 m a.s.l. (range 74-377 m a.s.l.), and exposed mainly to the south. The registered dog population in 2013 consisted of 119 animals, with an estimated 5% of unregistered dogs, for a total of about 125 dogs, and a similar estimation of 127 dogs in 2017 (data provided by Baone municipality).

### 2.2. Field Sampling

In Calaone, two new sampling campaigns were organized at the beginning of June 2013 and at the end of May 2017. Dog owners were invited to test their dogs for leishmaniosis and to fill in a questionnaire regarding mainly the use of preventative measures on their animals against sand fly bites. Moreover, during summer 2017, an entomological survey was conducted to update the sand fly population density already monitored in previous years [6, 20].

Serum and EDTA blood samples were collected from each study dog and stored at refrigerated conditions (+4-8°C) for laboratory analyses. Dog owners were interviewed briefly on individual data of their dog/s (e.g., age, movements to *Leishmania* endemic areas, sex, use, lifestyle, and previous serological tests) and more extensively on the use of preventive measures against sand fly bites during the previous summer seasons. The appropriateness of control measures was assessed and a dichotomous value (“correct” or “not correct”) was assigned to each dog, evaluating the used principle (if active against sand flies), the frequency of application (if following precisely the product's instructions), and the period of application (if corresponding to sand fly presence in the area: late June up to mid of September).

As per entomological survey, sand fly density was assessed in 2017 in two sites (namely, CE1 and CE3), located in Calaone and already monitored for sand flies in previous years [6, 20]. Site CE1 was monitored during the years 2006 and 2007 (hereafter considered jointly as Period 1) and in 2009 and 2010 (Period 2). Site CE3 was previously monitored only in Period 2. The number of entomological samplings was limited to 7 per year (with a 2-week interval), covering an identical period for all sampled years (starting date between 4 and 8 July; ending date between 28 September and 5 October). Each sampling was performed using 10 sticky traps (paper sheets of 20 × 20 cm coated with castor oil), which were left hanged at a height of approximately 1.5 m above the ground for a single night (from 19:00 to 07:00).

### 2.3. Laboratory Analyses

#### 2.3.1. Blood Samples

Serum was separated by centrifugation (3000 rpm for 5 min) from each blood sample and was submitted to the immunofluorescence antibody test (IFAT) according to OIE Manual of Diagnostic Tests and Vaccines for Terrestrial Animals [21]. IFAT was considered positive for serum titres ≥ 1 : 40.

EDTA-blood samples were stored at -20°C for molecular analyses. Blood samples of IFAT-positive dogs of both campaigns were submitted to a real-time PCR to detect DNA of circulating *Leishmania infantum*.

For DNA extraction, the NucleoSpin® Tissue Kit (Macherey-Nagel, GmbH & Co. KG, Germany) was used according to the manufacturer's instruction. Then, the extracted DNA was amplified using a SYBR® Green Real-Time PCR targeting a kDNA gene (230 bp) with the forward primer FLC2 (5′-GTCAGTGTCGGAAACTAATCCGC-3′) and the reverse primer RLC2 (5′-GGGAAATTGGCCTCCCTGAG-3′) designed by Gualda et al. [22]. The cycling conditions, performed in a 7900HT thermocycler (Applied Biosystems, California, USA), were as follows: the final reaction volume was 20 *μ*l with 10 *μ*l QuantiFast™ SYBR® Green PCR Master Mix 2x concentrated (Qiagen, Germany), 6.6 *μ*l RNase-free water, 0.2 *μ*l (0.1 *μ*mol) of each specific primer, and 3 *μ*l of the DNA isolated from the blood samples. PCR amplification consisted of 95°C for 5 min followed by 40 cycles at 95°C for 15 s, then 58°C for 30 s, and 60°C for 30 s. After the amplification cycle, positive samples were detected using the melting curve analysis. The temperature was increased slowly from 60 to 95°C for 15 s at a rate of 0.1°C s^−1^ with continuous monitoring of fluorescence. The specific melting temperature (*T*_m_) was registered for each amplified sample. In each reaction, positive (*L. infantum* DNA) and negative (no DNA) samples were added.

#### 2.3.2. Sand Fly Specimens

All the collected sand flies were counted, stored in 70% ethanol, and then observed under a microscope for gender separation and identification up to species level, according to morphological features [23].

The phlebotomine abundance, expressed as number of sand flies/m^2^/day, was evaluated counting the sand flies collected by sticky traps (i.e., 25 sheets covered an area of 1 m^2^).

### 2.4. Statistical Analyses

Differences in seroprevalence among the current and the previous campaigns were evaluated by Pearson's chi-squared test.

Differences in sand fly population densities (sticky trap data) among the three periods investigated (i.e., Period 1 = years 2006-2007; Period 2 = years 2009-2010; and Period 3 = year 2017) were evaluated by the Mann-Whitney *U* test (pairwise comparison) and Kruskal-Wallis tests.

The statistical analyses were performed by SPSS Statistics software, version 22.0.0 (IBM®, New York, USA). The acceptable level of significance was set at *p* < 0.05.

## 3. Results and Discussion

### 3.1. Epidemiological Survey

The northward spread of CanL and the emerging of new foci in previously free areas of Europe [4] moved public health authorities to pay major attention on prophylaxis and control. In an outbreak, the main goal is the reduction of pathogen circulation, limiting the contact between infected dogs and vectors.

The literature suggests that the prevention on dogs is more effective when matching more preventive measures, i.e., mechanical, chemical, and immunological tools [10]. Mechanical tools are represented by keeping dogs indoors during the sand fly season from sunset to sunrise and the use of small-meshed mosquito nets; chemical measures include different types of topical insecticides on dogs with proven activity against the sand flies such as spot-on, collars, and spray containing permethrin or synthetic pyrethroids [12–16]; immunological measures are represented by vaccines and two formulations are recently available in Europe [17, 18]. In Italy, recent studies proved that the massive use of topical insecticides protects dogs from the infection [12, 15, 24], but all these studies were conducted under experimental and controlled conditions. On the contrary, an apparent similarity infection rate between dogs with and without drugs active against ectoparasites was recently reported in an epidemiological survey conducted in Lampedusa island (southern Italy) [25]. However, the investigated area is a hyperendemic one and authors could not obtain detailed information about the compounds nor regarding the composition of the applied substances. To our knowledge, our study is the first report in Italy of a successful control of a new stable leishmaniosis outbreak through the massive use of various topical insecticides applied by dog owners in outdoor and uncontrolled conditions.

**Figure 1:**
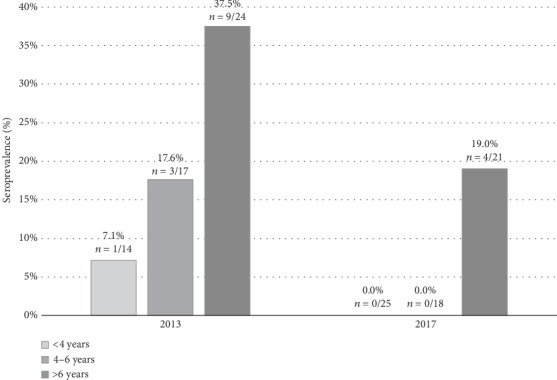
Canine seroprevalence values distributed in age classes (<4, 4-6, and >6 years) in 2013 (*n* = 55) and in 2017 (*n* = 64).

A total of 55 dogs were sampled in 2013 and 64 in 2017, corresponding to the 44% and the 50% of the total estimated canine population, respectively. The seroprevalence in canine population was 23.6% (*n* = 13/55) in 2013 and 6.2% (*n* = 4/64) in 2017. In 2013, the higher seroprevalence was recorded among dogs older than 6 years and this data was confirmed in 2017 when all positive dogs were older than 6 years (Figure 1). Most of the IFAT-positive dogs presented serum titre near the threshold (1 : 40 or 1 : 80) and were PCR negative, with the exception of one dog with serum titre of 1 : 2560, which was also PCR positive. The amplified DNA was sequenced and identified as *Leishmania infantum*.

**Figure 2:**
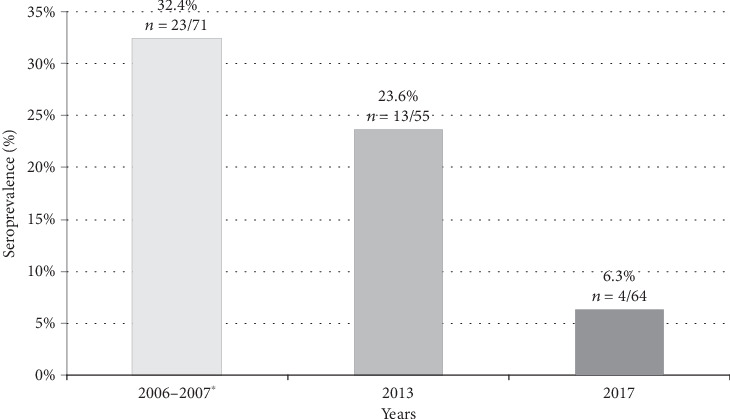
Comparison among seroprevalence values in dog population from 2006 until 2017. ^∗^Data previously published [8].

Comparison between seroprevalence values in dog population at the beginning of the survey in 2006-2007 (data previously published [8]) and in the two sampling campaigns of the present study (2013 and 2017) is reported in Figure 2. Difference in canine seroprevalence values between the last (2017) and the first (2006-2007) sampling was statistically significant (chi squared = 14.4, *p* value < 0.001). This clear decreasing trend (Figure 2) suggests that the circulation of the parasite decreased dramatically during the last decade. The four subjects found positive in 2017 were among the oldest dogs in the investigated population, since they were 10 years old or more (i.e., 10, 11, 12, and 17 years old) and were most probably exposed to infected vectors before the mass adoption of preventive measures by local dog owners.

**Figure 3:**
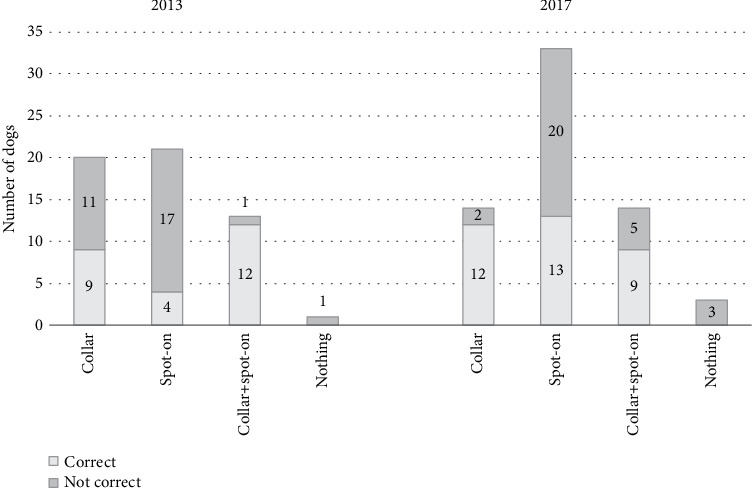
Distribution of correct/not correct use of preventive measures, as per declaration of dog owners in 2013 (*n* = 55) and 2017 (*n* = 64).

Most of the dog owners, 54/55 (98%) in 2013 and 61/64 (95%) in 2017, were well informed on CanL and on the need to prevent the infection and declared to have adopted preventive measures against sand fly bites (i.e., collar, spot-on, spray, or a combination of two/more of them) in the previous summer seasons (Figure 3). At the same time, collected data highlighted that insecticides were not always correctly used as shown in Figure 3 (i.e., used principle was not effective against sand flies and were not used as frequently as suggested by the manufacturer, the period of use does not cover entirely the sand fly season), resulting in an incomplete protection. However, notwithstanding this reduced protection of individual dogs, the common action of use of topic insecticides on dogs adopted by local owners in the last ten years appeared to be effective in reducing the circulation of the parasite.

### 3.2. Sand Fly Samples

**Figure 4:**
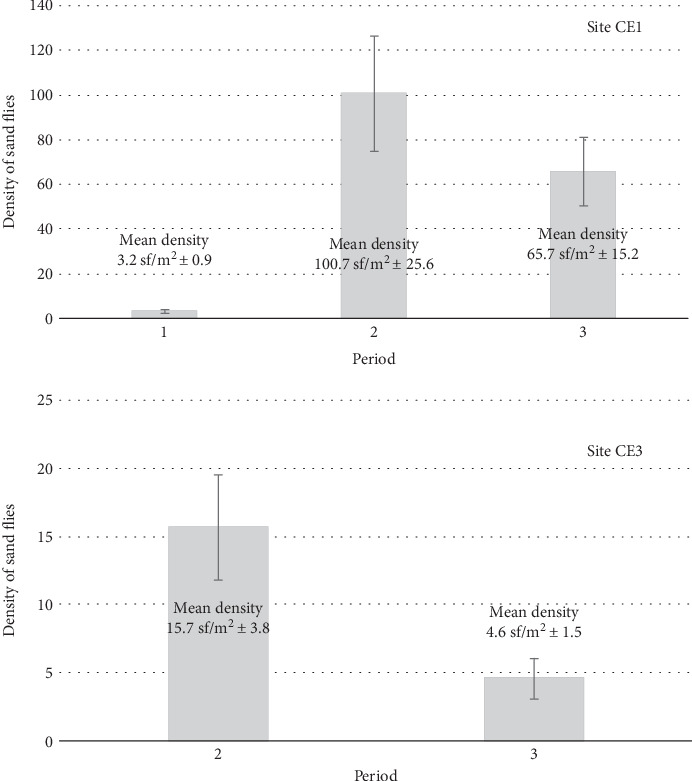
Bar plot representing sand fly (sf) densities (number sf/m^2^ per day) in sites (a) CE1 and (b) CE3, in the three periods (Period 1: 14 samplings; Period 2: 14 samplings; and Period 3: 7 samplings). The mean density values and their standard error (±SE) are also reported.

A total of 195 sand flies were captured in 2017. Among the collected specimens, 81.2% (160/195) were *Phlebotomus perniciosus* and 4.1% (8/195) *Phlebotomus neglectus*, whereas for the remaining it was not possible to reach a specific identification. The average density of phlebotomine sand flies was 65.7 and 4.6 sand flies/m^2^ per day in sites CE1 and CE3, respectively. A mean density of 3.2 sand flies/m^2^ per day was recorded in Period 1 and 100.7 in Period 2 in site CE1 (unpublished data). In site CE3, monitored only during Period 2, a mean density of 15.7 sand flies/m^2^ per day was recorded. The comparison of sand fly densities in site CE1 highlighted significant difference among periods (Kruskal-Wallis = 17.3, *p* value < 0.001) and specifically between Periods 1 and 2 (Mann-Whitney *U* = 24, *p* value < 0.001) and between Periods 1 and 3 (Mann-Whitney *U* = 11.5, *p* value = 0.003), whereas no significant change was found between Periods 2 and 3 (Mann-Whitney *U* = 41, *p* value = 0.585).  Figure 4 shows how sand fly density significantly increased in the period (2006-2010) corresponding to the initial phase of CanL spread and remained high in the subsequent period (2010-2017), despite an apparent decrement. The similarity in density values and the partial reduction between Periods 2 and 3 (Figure 4) were also confirmed by comparing samplings of site CE3 (Mann Whitney *U* = 24, *p* value = 0.067). Notwithstanding period, time frame and frequency of samplings in the different years were planned in a way to make them as much comparable as possible; also, differences in climatic parameters (e.g., temperature, rainfall) may have influenced sand fly densities. However, the finding that only a limited decrement (without significant variations) was recorded in sand fly abundance since the period 2009-2010 suggests that the decrease in dogs' seroprevalence was mostly due to a reduction of the contact between the vector and definitive host.

Another option to evaluate the significance of sand fly in *L. infantum* transmission consisted in the determination of the infection rate in collected specimens. In the present study, it was not possible to assess this parameter, partially due to the limited number of female insects that were eventually available for biomolecular analysis.

## 4. Conclusions

This study concludes a survey in a CanL outbreak in a hilly area of northeastern Italy. During this long period, the canine population was serologically monitored, but the main action for the outbreak control was the sensitization of local citizens about CanL. This survey demonstrated that the action conducted at the beginning and during the outbreak greatly helped to reduce the impact of CanL. Undoubtedly, the fact that the focus occurred in a small village with few dogs helped to reach many owners with the correct information. The approach adopted during the intervention and the sensitization activity jointly performed by researchers, local authorities, and private veterinarians represent a good example of collaboration, able to achieve excellent results in the management of a CanL outbreak.

## Data Availability

The authors declare that data are available on request to the corresponding author by email.
